# Designer immune cells

**DOI:** 10.1186/s41232-024-00362-1

**Published:** 2024-11-25

**Authors:** Naoki Hosen

**Affiliations:** Department of Hematology and Oncology, Osaka University Graduate School of Medicine, 2-2, Yamada-Oka, Suita, Osaka, 565-0871 Japan

Various types of immune cells can be genetically engineered to perform any function. The resulting therapeutic cells are called “designer immune cells.” The prototype of designer immune cell therapy is CAR T-cell therapy. CD19 CAR T-cell therapy targeting CD19 or BCMA has demonstrated overwhelming therapeutic efficacy in patients with B-cell leukemia/lymphoma or multiple myeloma [1, 2]. The advent of CAR-T is a historic turning point that has demonstrated that “cell” modalities can be established as a revolutionary treatment modality. The cell types used for designer immune cells are not only T cells. NK cells, macrophages, or iPS-derived immune cells are now being tested as sources of designer immune cells. In addition, transduction of conventional CAR constructs is not the only way to confer anti-tumor function to these cells. Several types of synthetic receptors are now being tested to generate new designer immune cells.

In this thematic series of reviews, we have invited the leading researchers in this field. Dr. Kaneko and colleagues reviewed their series of original studies on iPSC-derived CD8 killer T cells, drawing insights to overcome the exhaustion associated with antigen-specific T-cell therapy. Dr. Moroishi and colleagues summarized their series of original studies regarding on the molecular mechanisms by which the Hippo pathway controls cell–cell communication and discussed its importance in tissue homeostasis. I reviewed the literature on target antigens for CAR T-cell therapy and presented our work on identifying novel targets for hematological cancers. Dr. Toda and colleagues presented recent advances in synthetic receptor technology, which is key to designing therapeutic cells that improve the tumor microenvironment or regenerate damaged tissues. Dr. Kagoya and colleagues reviewed recent findings on molecular insights into T-cell dysfunction and how genetic modification contributes to overcoming it. They also discussed several technical advances to achieve efficient gene modification using CRISPR and other novel platforms. I would like to express my sincerest gratitude to the esteemed researchers who have contributed to this special issue. It is my sincere hope that these review articles will provide new insights to researchers engaged in the broad field of inflammation and regeneration.
